# Twin delivery: does induction of labor make a difference?

**DOI:** 10.1007/s00404-025-07939-2

**Published:** 2025-03-14

**Authors:** L. Rüegg, L. Vonzun, J. Wawrla-Zepf, F. Krähenmann, N. Ochsenbein-Kölble

**Affiliations:** 1 Department of Obstetrics, University Hospital Zurich, Rämistrasse 100, 8091 Zurich, Switzerland; 2 The Zurich Center for Fetal Diagnosis and Therapy, University of Zurich, Zurich, Switzerland; 3 University of Zurich, Rämistrasse 71, 8091 Zurich, Switzerland

**Keywords:** Twins, Twin delivery, Mode of delivery, Induction of labor, Intertwin interval

## Abstract

**Purpose:**

Induction of labor as well as delivery in twin pregnancies bears specific risks. The goal of this study was to analyze the delivery mode in twin pregnancies and influence of induction on the cesarean delivery (CD) rate and perinatal outcome and to identify risk factors for CD and an intertwin interval ≥ 15 min.

**Methods:**

This single-center retrospective cohort study analyzed the outcome of 267 twin pregnancies. Inclusion criteria for vaginal delivery in twins are gestational age (GA) > 34 weeks, leading twin in cephalic presentation, estimated weight difference ≤ 500 g and no previous uterine surgery. Women were divided into three groups: 1 = vaginal delivery, 2 = CD for both twins, 3 = emergency CD for second twin. Outcomes were mode of delivery, and influence of induction on the CD rate.

**Results:**

We had 156 women (58%) in group 1, 97 (36%) in 2 and 14 (5%) in 3. Induction of labor was performed in 147 cases and led to a higher CD rate (23% vs. 47%, *p* < 0.001). Induction of labor and nulliparity were associated with a higher risk for CD for both twins. Risk factors for an intertwin interval of ≥ 15 min were maternal age and weight of the second twin. An intertwin interval of ≥ 15 min was associated with a lower umbilical artery pH in the second twin.

**Conclusion:**

The rate of CD doubled if induction of labor was necessary. These results emphasize on careful patient counseling that includes information about the risks of deliveries in twin pregnancies.

## What does this study add to the clinical work

**Table:** 

Induction in twin pregnancies can increase the rate of cesarean delivery. In this single center cohort study the delivery mode of twins was analyzed and the results can influence counselling women expecting twins to make an informed decision about their chosen delivery mode and about possible risks of induction of labor and risks for the second twin.	

## Introduction

The prevalence of twin pregnancies has doubled in many countries since 1970 mainly due to the rising maternal age and increased use of reproductive medicine [1–3].

Twin pregnancies are at risk for various complications such as preeclampsia, growth restriction or intrauterine fetal demise [4–7]. They have a higher fetal and maternal morbidity and mortality rate [8], especially near term. Thus, several guidelines recommend elective delivery of uncomplicated dichorionic twins around 37–38 gestational weeks (GW) and at around 37 GW in monochorionic twins [9–14].

Whether induction of labor in twins leads to a higher cesarean delivery (CD) rate is still under debate [15–20]. During vaginal delivery, the second twin faces a higher risk than the first [21, 22].

After successful delivery of the first twin, insufficient contractions, malpresentation, placental disruption or prolapse of the umbilical cord can complicate the delivery of the second twin, and a manual extraction, vaginal operative measurements or even a delivery via CS for the second twin can become necessary [23]. The ideal time frame, in which the second twin should be delivered, is still under discussion. Several studies demonstrated a longer intertwin interval to be associated with an increased morbidity of the second twin [24–26]. Therefore, it is often recommended to deliver the second twin within 15 min after the first [27].

The primary aims of this study were to evaluate delivery mode in twins at our center and analyze whether induction of labor increases the frequency of CD for both twins or of emergency CD for the second twin. The secondary aim was to identify risk factors for CD of both twins, emergency CD in the second twin or an intertwin interval of ≥ 15 min.

## Methods

### Patients and study design

This is a retrospective single-center cohort study.

Between 2004 and 2022, a total of 2628 twin deliveries took place at our tertiary center. Unfortunately, 1706 were ruled out due to missing informed consent. Of the remaining 922 pregnancies, 321 women had a scheduled primary CD. Indication for primary CD in twins at our centers includes leading twin not in cephalic position, leading twin > 500 g smaller than the second twin, previous CD in medical history or mothers wish due to the twin pregnancy. Further, we excluded preterm deliveries before 34 weeks (*N* = 116) because we only deliver twins vaginally after 34 weeks at our center. Also, women with delivery due to the beginning of labor before scheduled CD (*N* = 148) or with loss of pregnancy (*N* = 70) were excluded from this study.

Criteria for a vaginal delivery in twins at our center were as follows: gestational age (GA) > 34 weeks, leading twin in cephalic presentation weighing at least 2000 g, estimated birth weight difference ≤ 500 g, no previous uterine surgery. Induction was performed due to maternal or fetal indications such as GA (until 2018 all twins were induced after completing 38 weeks since 2018 uncomplicated dichorionic–diamniotic twin pregnancies are induced between 37–38 GW and monochorionic–diamniotic pregnancies around 37 GW according to the recommendations for management of twin pregnancies of the German, Austrian and Swiss Society for obstetrics and gynecology [11], premature rupture of membranes (PROM) or preterm premature rupture of membranes (PPROM), oligohydramnios, intrauterine growth restriction (IUGR), mild preeclampsia, pregnancy hypertension, suspicious cardiotocography (CTG) pattern, cholestasis of pregnancy or also the wish of the mother.

Induction in twin pregnancies was performed according to the induction protocol of our department with intravenous application of oxytocin for 6 h (5 IE in 500 ml) with a running rate starting at 12 ml/h. Dose was increased by 12 ml/h every 30 min until regular contractions (4/10 min) were present. After 6 h, a break of at least 4 h was mandatory before starting the next cycle. If unsuccessful, either the insertion of a uterine balloon (Cook-Balloon®) for 24 h (or until dislocation) or the application of vaginal misoprostol (misoprostol 25 µg, every 4–6 h, up to a maximum of 6 doses) was discussed and applied on patient’s agreement. When patients refused induction and decided for primary CD, they were excluded from this study cohort. Indication for CD was an individual decision in each case depending on the mother’s and children’s well-being, the delivery course and the mother’s wish.

Baseline data as maternal age, maternal body mass index (BMI), parity, conception and chorionicity were retrieved from our electronic database. Further, mode of delivery for both twins, induction, reason for induction, intertwin interval and neonatal characteristics, such as birthweight, umbilical artery pH and Apgar, were analyzed.

We then divided the twin deliveries in three groups as follows: Group 1: vaginal delivery of both twins, group 2: unplanned CD during labor (due to fetal compromise, labor dystocia, failed induction, etc.) for both twins, group 3: an emergency CD for the second twin after vaginal delivery of the first twin. We compared the outcomes between these three groups, as well as the influence induction has on the delivery mode. For the comparison of induction, we divided the women in an induction-group and a no-induction group. For the secondary outcome, we analyzed possible influencing factors on intertwin delivery interval in each group.

### Data analysis

Descriptive statistics were performed with SPSS Statistics (version 27, IBM, USA).

The distribution of the data was analyzed using Shapiro–Wilk Test. Depending on the distribution of the data, it is presented as mean ± standard deviation (SD) or median with interquartile range (IQR, 25% and 75%). Categorical variables are presented as numbers (N) with percentages (%). Groups were compared using Kruskal–Wallis’s test, Pearson’s chi-squared test or Fisher’s exact test, as appropriate. Binary regression analysis was performed to identify risk factors for CS or an intertwin interval of ≥ 15 min and a multiple regression analysis was performed. Statistical significance was given with *p* < 0.05.

The study was conducted according to the principles of the Declaration of Helsinki and the International Conference on Harmonization E6 (Good Clinical Practice) guidelines. All women included signed a general informed consent. The study was conducted with the approval of the local Ethic Commission (KEK-ZH Nr. 2022–01356).

## Results

We included 267 women with twin pregnancies in this study. Baseline characteristics of our cohort are presented in Table 1.

**Table 1:** Baseline characteristics of the study cohort

Baseline characteristics	*N* = 267
Maternal age, [years], median (IQR)	33 (30–37)
Maternal BMI, [kg/m^2^], median (IQR)	21.6 (19.8–23.9)
Nulliparous women, [*N* (%)]	144 (54)
Natural conception, [*N* (%)]	175 (66)
Dichorionic–diamniotic twins, [*N* (%)]	198 (74)
Monochorionic–diamniotic twins, [*N* (%)]	67 (25)
Unknown chorionicity, [*N* (%)]	2 (1)

*GA* = gestational age, *GW* = gestational weeks

### Mode of delivery in twins

Of the 267 women who planned a vaginal delivery, 156 women delivered vaginally (58.4%; (group 1)), while 97 had an unplanned CD during labor for both twins (36.4%; (group 2)), mostly due to labor dystocia or failed induction (Table 2). An emergency CD for the second twin was necessary in 14 cases (5.2%; (group 3)) with the main reason being fetal compromise followed by malpresentation. Induction of labor was performed in 72 women (46%) in group 1, and in 71 (73%) and 6 (43%) in group 2 and 3, respectively. When an emergency CD had to be performed for the second twin, as in group 3, their 5-min Apgar score and umbilical artery pH were significantly lower than in group 1 and 2 (Table 2).

**Table 2:** Comparison of delivery mode in twin gestations

	Group 1 (*N* = 156)	Group 2 (*N* = 97)	Group 3 (*N* = 14)	*p*-value
Nulliparous women, [*N* (%)]	**64 (42)**	**76 (78)**	**4 (29)**	** < 0.001**
Induction of labor, [*N* (%)]	**72 (46)**	**71 (73)**	**6 (43)**	** < 0.001**
Chorionicity, [*N* (%)]				
Dichorionic–diamniotic	128 (82)	74 (76)	11 (79)	
Monochorionic–diamniotic	28 (18)	23 (24)	3 (21)	0.54
GA at delivery, [gw]	37.4 (36.3–28.1)	37.4 (36.6–38.3)	36.9 (36.6–37.8)	0.33
Reason for CD, [*N* (%)]**				
Failed induction		25 (26)		
Labor dystocia		47 (49)		
Fetal distress		16 (17)		
Malpresentation		2 (2)	7 (50)	
Others		6 (6)	4 (29)	
Birth weight, [g]				
Twin A, mean ± S	2641 ± 369	2649 ± 403	2581 ± 447	0.61
Twin B, median (IQR)	2595 (2258–2845)	2560 (2270–2850)	2560 (2378–2730)	0.82
5 min Apgar score, median (IQR)				
Twin A	9 (9–9)	9 (9–9)	9 (9–9)	0.05
Twin B	**9 (8–9)**	**9 (9–9)**	**8 (7–9)**	** < 0.001**
PH, median (IQR)				
Twin A	7.30 (7.25–7.34)	7.31 (7.29–7.34)	7.30 (7.28–7.34)	0.05
Twin B	**7.26 (7.20–7.30)**	**7.31 (7.27–7.34)**	**7.18 (7.14–7.27)**	** < 0.001**

**For emergency CD for the second twin; data only available on 11 cases, *GA* = gestational age, *GW* = gestational weeks; *SD*, standard deviation

Statistical significance was given with *p* < 0.05. All statistically significant results were marked bold

When analyzing and comparing risk factors for CD among the groups 1–3, results showed that significantly more women in group 2 were nulliparous (p < 0.001) and underwent induction of labor (*p* < 0.001) (Table 2).

### Induction of labor and its influence of the mode of delivery in twins

Reasons for induction are presented in Table 3. The most common reason for induction was GA (70%) followed by mothers’ wish (8%) and PROM/PPROM and preeclampsia (7% both). In the induction group, GA at delivery and birth weight was significantly higher than in the no induction group (GA: *p* < 0.001, birth weight: *p* < 0.001; Table 3). When induction of labor was performed, the CD rate for both twins doubled (23% with no induction vs. 47% with induction, *p* < 0.001). There was no increase in emergency CD for the second twin after induction of labor, no differences were observed regarding the neonatal outcome in the induction and no-induction group. Apgar and pH were comparable between both groups (Table 3).

**Table 3:** Comparison of delivery mode and neonatal outcome after induction of labor in twin gestation

	No Induction (*N* = 118)	Induction (*N* = 149)	*p*-value
GA at delivery, [GW]	**36.4 (35.3–37.4)**	**38.0 (37.3–38.4)**	** < 0.001**
Nulliparity, [*N* (%)]	64 (53)	80 (54)	1.0
Chorionicity, [*N* (%)]			
Dichorionic–diamniotic	98 (83)	115 (77)	
Monochorionic–diamniotic	20 (17)	34 (23)	0.24
Reason for induction [*N* (%)]*			
GA		93 (69)	
Mothers’ wish		11(8)	
Preeclampsia		10 (7)	
PROM/PPROM		10 (7)	
Oligohydramnios		6 (4)	
IUGR		2 (2)	
Suspicious CTG		2 (2)	
Cholestasis		1 (1)	
Delivery mode [*N* (%)]			
Group 1	**85 (70)**	**72 (49)**	** < 0.001**
Group 2	**26 (23)**	**71 (47)**	** < 0.001**
Group 3	8 (7)	6 (4)	0.41
Birth weight, [g]			
Twin A, mean ± SD	**2514 ± 370**	**2770 ± 365**	** < 0.001**
Twin B, median (IQR)	**2420 (2085–2713)**	**2700 (2410–2920)**	** < 0.001**
5 min Apgar, median (IQR)			
Twin A	9 (9–9)	9 (9–9)	0.97
Twin B	9 (8–9)	9 (9–9)	0.51
pH, median (IQR)			
Twin A	7.30 (7.27–7.33)	7.31 (7.27–7.34)	0.15
Twin B	7.27 (7.2–7.31)	7.28 (7.24–7.32)	0.17

*GA* gestational age, *GW* gestational week, *(P)PROM* (preterm) premature rupture of membranes, *IUGR* intrauterine growth restriction, *CTG* cardiotocography

*N = 135 (missing data of other women)

Statistical significance was given with *p* < 0.05. All statistically significant results were marked bold

The rate of vaginal delivery (*p* = 0.91), CD (*p* = 0.63) and emergency CD (*p* = 0.34) after induction of labor did not change after implementing the new protocol in 2018.

A univariate regression analysis identified induction of labor (*p* < 0.001), conception (*p* = 0.05), parity (*p* < 0.001) and nulliparity (*p* < 0.001) as possible independent risk factors for CD in twin deliveries. The multiple regression analysis then revealed only nulliparity and induction of labor to be independent risk factors for CD (*P* < 0.001 and *p* < 0.001 respectively, Table 5). Neither the reason for induction nor the chorionicity had influence on the mode of delivery.

An intertwin interval of ≥ 15 was found significantly more often in cases where induction was performed (*p* = 0.03). Generally, an intertwin interval > 15 min was found to be related with a significant lower umbilical artery pH in the second twin (< 15 min: 7.27 (7.22–7.31), ≥ 15 min: 7.21 (7.16–7.27), *p* = 0.01) (Table 4). The univariate regression analysis identified induction (*p* = 0.04), maternal age (*p* = 0.03) and birth weight of the second twin (*p* = 0.005) as possible risk factors for an intertwin interval of ≥ 15 min. The multiple regression analysis then identified higher maternal age and weight of the second twin to be the sole risk factor for an intertwin interval of ≥ 15 min or more (Table 5).

**Table 4:** Neonatal outcome according to intertwin interval with a cut-off of 15 min

	Intertwin Interval < 15 min (*N* = 99)	Intertwin Interval ≥ 15 min (*N* = 57)	*p*-value
Nulliparous women, [*N* (%)]	40 (40)	24 (42)	0.87
Induction of labor, [*N* (%)]	**39 (39)**	**33 (58)**	**0.03**
GA at delivery, [GW]	37.0 (35.9–37.9)	37.9 (36.9–38.3)	0.08
Birth weight, [g]			
Twin A, mean ± SD	2633 ± 380	2732 ± 422	0.08
Twin B, median (IQR)	**2490 (2210–2810)**	**2730 (2500–2940)**	**0.03**
5 min Apgar, median (IQR)			
Twin A	9 (9–9)	9 (9–9)	0.90
Twin B	9 (8–9)	9 (8–9)	0.61
pH, median (IQR)			
Twin A	7.30 (7.27–7.33)	7.30 (7.23–7.34)	0.63
Twin B	**7.27 (7.22–7.31)**	**7.21 (7.16–7.27)**	**0.02**

*GA* gestational age, *GW* gestational week

Statistical significance was given with *p* < 0.05. All statistically significant results were marked bold

**Table 5:** Multiple logistic regression analysis to predict influencing factors on CD rate and intertwin interval

	OR (95% CI)	*p*-value
Cesarean delivery		
Induction of labor	3.7 (2.05–6.66)	< 0.001
Nulliparity	6.5 (3.54–12.0)	< 0.001
Intertwin Interval ≥ 15 min		
Maternal age	1.1 (1.01–1.18)	0.02
Birth weight twin B	1.0 (1.0–1.002)	0.02

*OR* odds ratio, *CI* confidence interval

## Discussion

This study analyzed twin deliveries during the last 20 years and focused on whether induction of labor has an impact on the delivery mode in twin pregnancies. When planning a vaginal delivery, almost 60% of women delivered their twins vaginally. Induction of labor doubled the rate of CD in our cohort. Nulliparity and induction of labor were identified as risk factors for CD. No risk factors were identified for emergency CD for the second twin. Furthermore, emergency CD for the second twin was associated with a lower Apgar score and pH for the affected twin.

### Delivery in twin gestations

The Twin Birth Study [28] compared the risk for fetal or neonatal death or serious morbidity in twin pregnancies between 32 and 38 GW in either planned CD or planned vaginal delivery. Around 56% of women, with planned vaginal birth, delivered vaginally, while 40% had a CD for both twins and 4% experienced an emergency CD for the second twin [28]. These numbers are comparable to those in our cohort.

In a further large retrospective cohort study by de Castro et al. [29], 883 women with twin gestations underwent a trial of labor. The rate of vaginal delivery in this study was almost 87% [29]. One noticeable difference to our cohort is that their number of labor induction was 33%, while ours was 55%. This might be a reason why the numbers are difficult to compare between ours and their study. In line with our results, they found nulliparity to be a risk factor for CD in twin gestations [29].

### Induction in twin gestations

A retrospective observational cohort study was conducted by Lopian et al. [19] to determine and compare the safety and efficacy of induction of labor in twins. They compared women with a twin gestation > 32 GW that either underwent induction of labor (*N* = 268) or went into labor spontaneously (*N* = 450). Primary outcome was the CD rate. They found a twofold increased risk of CD in the group with induction of labor, while the method of induction (prostaglandins vs. oxytocin) did not make a difference [19]. Like in our cohort, induction of labor was associated with a higher rate of CD.

Another cohort study, from two University Hospitals in Sweden, investigated the association between induction of labor and CD [20]. They compared twin pregnancies with induction of labor (*N* = 220) and spontaneous onset of labor (*N* = 242) in twin pregnancies. Again, they found a significant higher rate of CD in the induction group (21 vs. 12%) [20]. In their cohort, 80% of women with induced labor delivered vaginally. These results clearly differ from ours since the rate of vaginal deliveries after induction was only 52%.

Comparison of the indications for induction and for secondary CD is difficult and might add to some of this difference. For example, how many women wish to proceed with delivery by CD instead of continuing with induction is unknown. Additionally, 68% of the inductions in the Swedish cohort were started by amniotomy, a method that we do apply however not that often [20].

Two studies, including a total of 435 twin pregnancies, compared expectant management with induction of labor and both found no increased rate in CD [15, 30].

To conclude, data about induction and rate of CD in twin pregnancies remains somewhat controversial. Yet, in our cohort, we could show that induction of labor leads to a higher risk for CD. Implementing the new protocol in 2018, with a recommendation of induction of labor at 37 weeks for monochorionic twins and 37–38 weeks in dichorionic twins [11], did not further increase the CD rate after induction of labor.

Ultimately, this demands for valid indications for induction but also for CD. When counseling patients regarding the mode of delivery in twin pregnancies, risks of each method need to be evaluated and discussed.

### Emergency CS for the second twin

A study of Aviram et al. [31] examined the incidence, risk factors and outcomes of CD for the second twin (combined delivery) [31]. Their overall rate of a vaginal delivery in the first twin, in women who planned a vaginal birth, was slightly higher than in our cohort (60.4% vs. 51%). In their cohort, in 7%, the second twin was delivered via CD, which is comparable to our data. The most common reasons for CD in the second twin were malpresentation, fetal compromise and cord presentation / prolapse, which are similar to our findings, where fetal compromise and malpresentation were the most common reasons for CD in the second twin. They also reported a higher neonatal morbidity (5-min Apgar score < 7, neonatal intensive care unit admission, abnormal level of consciousness and prolonged assisted ventilation) after CD for the second twin compared to vaginal delivery in both twins [31].

### Intertwin interval, pH and Apgar score

After delivering the first twin, several problems (such as insufficient contractions, malpresentation, placental disruption or prolapse of the umbilical cord) can lead to difficulty in the delivery of the second twin, and therefore expose the second twin to a higher risk of hypoxia. In a study from McGrail et al. [32], 144 twin deliveries were analyzed which showed that both umbilical cord arterial and venous pH declined for the second twin as the interval between their delivery increased (arterial pH 0–15 min: 7.25 ± 0.06, 16–30 min: 7.22 ± 0.07). This is in accordance with our findings. But neither their nor our findings are clinically strong enough to influence or plea for a change of management. This is undermined by a retrospective study from Schneuber et al. which included 207 twin pairs. Their results did not show a negative impact on the second twin if the interval from delivering twin one to twin two exceeded 15 min [33].

Very strict intertwin intervals of 10 min as well as more ease intervals of 30 min have been looked at as well [34]. However, a clear definition of the ideal intertwin interval cannot be deduced from literature. Probably other factors, such as fetal wellbeing and course of delivery are more important than a precise time definition.

Taken together, vaginal delivery in twin births bears risks for the second twin. Moreover, women who choose to deliver vaginally need to be informed about the risks, especially for the second twin and about the possibility of an emergency CD for the second twin.

### Limitations and strengths

One limitation is the retrospective cohort study design. A randomized trial would be needed to definitively establish causality between induction and CD rates. Another is the high rate of exclusions due to missing informed consent. A further limitation might be that our inclusion criteria for vaginal twin delivery are not completely applicable to all other centers.

A strength of this study is that data of one single center for 20 years were analyzed including clear reasons for induction of labor in twins and reasons for CD and emergency CD, with documented chorionicity in almost all cases. The results of this study could be used in counseling pregnant patients who wish to deliver twins at our center.

## Conclusion

In about 60% of all planned vaginal deliveries in twin pregnancies, both twins were delivered vaginally, whereas the rate of CD for both twins was 36%. The CD rate doubled if induction of labor was performed. Nulliparity was an additional risk factor for CD. These results emphasize carefully considered indication for induction of labor as well as patients counseling that includes information about the different delivery modes and their associated risks. To fully establish causality between induction and higher CD rates, further studies, including a randomized trial, would be needed.

## Data Availability

The data which supports the findings of our study are not openly available due to reasons of privacy restrictions and sensitivity. The data are available from the corresponding author upon reasonable request.
